# Serum sodium within the normal range and its U-shaped relationship with biological aging in U.S. adults

**DOI:** 10.3389/fnut.2025.1589962

**Published:** 2025-05-08

**Authors:** Xianxiang Tong, Fei Wang, Mengxue Liu

**Affiliations:** Department of Anesthesiology, Sichuan Provincial People's Hospital, School of Medicine, University of Electronic Science and Technology of China, Chengdu, China

**Keywords:** serum sodium, biological age, hydration, aging, hydration management

## Abstract

**Background:**

This cross-sectional study evaluated the correlation between serum sodium levels (135–145 mmol/L) and biological aging in U.S. adults. Biological age, derived from multi-system biomarkers, provides a more accurate assessment of aging than chronological age. Hydration balance, reflected by serum sodium, may modulate age-related diseases and mortality, but its link to biological aging remains underexplored.

**Methods:**

Using NHANES data (1999–2018), we focused on normonatremic adults (≥20 years). The final cohort included 18,301 participants. Biological age was estimated using the Klemera and Doubal method, and ∆age (biological age—chronological age) was calculated. Associations were assessed using multivariate regression, generalized additive models, and threshold analysis. Subgroup analyses were conducted for variations across different populations.

**Results:**

Nonlinear analysis revealed a U-shaped relationship between serum sodium and biological age. The lowest biological age occurred at 139.3 mmol/L: each 1 mmol/L increase below this threshold was associated with a reduction of 0.10 years in biological age (95% CI: −0.15, −0.05), whereas values above it showed a 0.08-year increase (95% CI: 0.04, 0.13). For ∆age, a negative association was observed below 141.2 mmol/L, with each increase linked to a 0.07-year decrease (95% CI: −0.10, −0.04). Subgroup analyses revealed significant interactions in diabetic and smoking populations.

**Conclusion:**

Maintaining serum sodium levels within an optimal range (138–142 mmol/L) may help delay biological aging. Hydration management may serve as a modifiable factor for healthy aging, particularly in high-risk groups such as individuals with diabetes or tobacco use.

## 1 Introduction

As documented in the World Health Organization’s Global Health Observatory report (2019), worldwide longevity metrics demonstrated a surge of 6.3 years during the 21st century’s initial two decades, progressing from 66.8 years (2000) to 73.1 years (2019). However, healthy life expectancy (HALE) has not kept pace with this increase, highlighting a growing disparity between lifespan and health span. As the global population ages and the prevalence of age-related chronic diseases rises, identifying strategies to delay aging has become a critical focus in preventive medicine (1–3). Safe, practical, and widely applicable anti-aging interventions could not only slow aging and extend healthy lifespan but also improve quality of life and reduce healthcare costs more effectively than disease-specific treatments (4, 5).

Biological aging refers to the progressive decline in physiological function across multiple systems. While aging is inevitable, interindividual heterogeneity in its progression underscores the need for personalized biomarkers (6). Chronological age often fails to accurately reflect the rate of physiological deterioration. In contrast, biological age, which is derived from age-dependent biomarkers and clinical data, provides a more reliable measure of an individual’s aging trajectory, remaining lifespan, and susceptibility to age-related diseases (7, 8).

The connection between hydration and health outcomes has been examined in prior research. Suboptimal hydration has been associated with cognitive impairments (9), reduced physical performance (10), multisystem diseases (11), and even reduced life expectancy (12). It may accelerate cellular senescence through mechanisms involving oxidative stress, impaired proteostasis, and mitochondrial dysfunction, whereas optimal hydration could mitigate age-related telomere attrition and inflammatory pathways (13, 14). Plasma osmolality—maintained within the narrow range of 275–295 mosmol/kg—has been widely validated as a reliable and independent indicator of hydration status (15). However, due to its limited routine clinical application, indirect measures such as serum sodium, urine specific gravity, or urine color are commonly used to reflect hydration status. When hyperglycemia and renal failure are absent, serum sodium levels predominantly influence plasma osmolality (16, 17), establishing it as a key indicator of hydration status (18).

Evidence from previous cohort analyses suggests that serum sodium concentrations may serve as a predictive biomarker for morbidity and mortality. A cross-sectional analysis of adults aged 51–70 with serum sodium concentrations below 135 mmol/L or above 145 mmol/L demonstrated that inadequate hydration was linked to a higher likelihood of adverse health outcomes and mortality (19). Furthermore, a community-based study of 11,255 middle-aged adults (45–66 years) with normal serum sodium levels (135–146 mmol/L) showed that levels above 142 mmol/L were linked to faster aging, more chronic diseases, and early death (20). However, these studies focused on older or middle-aged populations and did not incorporate multidimensional biological aging metrics. To the best of our knowledge, no population-based studies have yet examined the association between serum sodium levels and biological aging among U.S. adults aged 20 years and above, despite the unique relevance of this population given widespread suboptimal hydration habits (15, 21), high sodium diets (22), and rising rates of metabolic disorders (23) that exacerbate hydration imbalances. To address this gap, we performed a cross-sectional analysis utilizing data from the National Health and Nutrition Examination Survey (NHANES) to explore the association between serum sodium concentrations and biological aging in a nationally representative cohort of U.S. adults.

## 2 Materials and methods

### 2.1 Study population

The NHANES is a nationwide cross-sectional survey conducted annually in the United States. It employs a complex sampling strategy to assess the population’s health and nutritional status. In this study, data from 10 cycles over 20 years (1999–2018) were analyzed.

Initially, 49,259 participants with available serum sodium and biological age data were included. Individuals below the age of 20 and pregnant women were excluded from the study. To ensure the absence of acute or chronic water-electrolyte imbalances, only participants with serum sodium levels falling within the normal range (135–145 mmol/L) were included.

**Figure 1:**
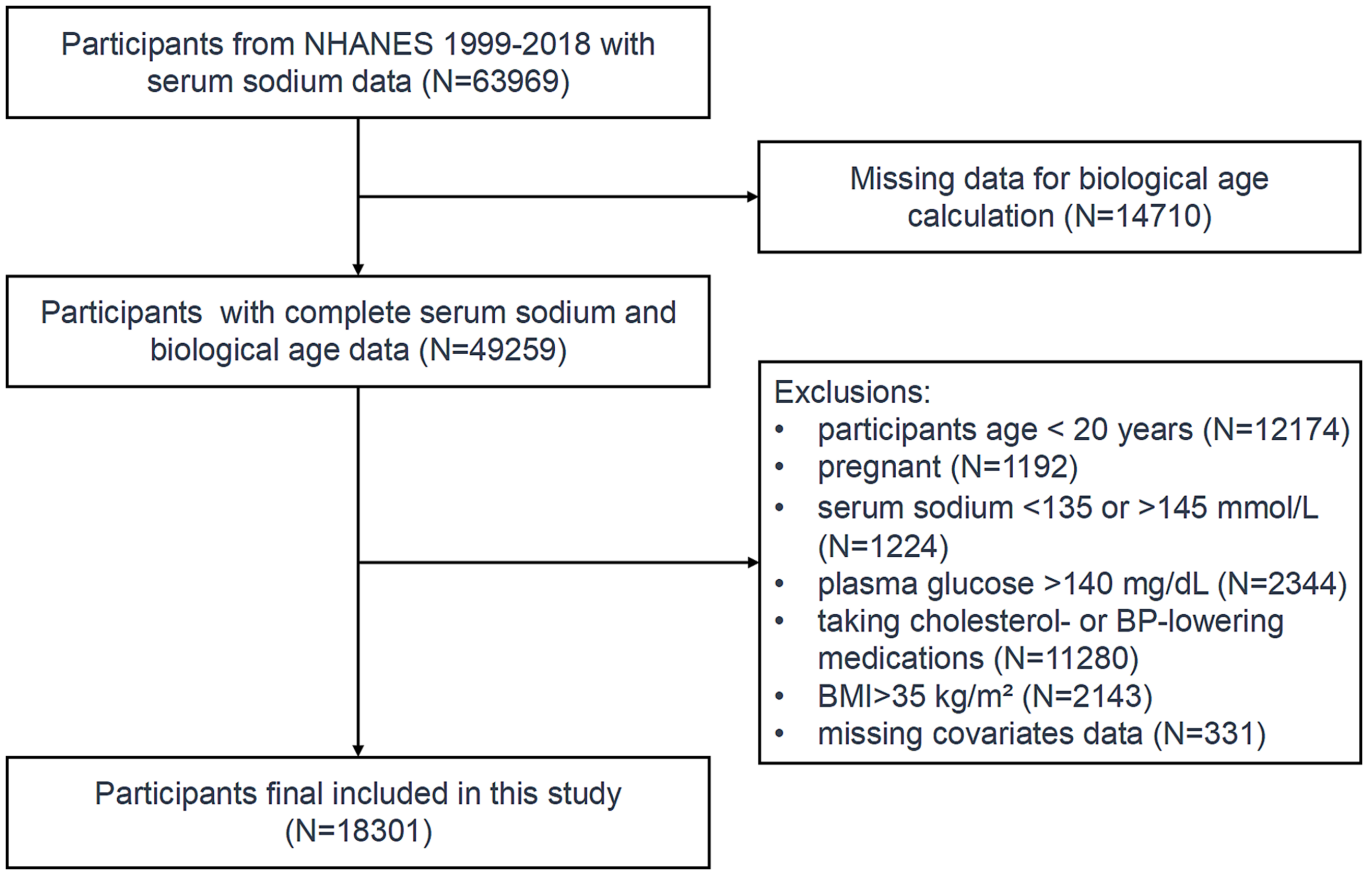
Participants inclusion flowchart.

Hyperglycemia may cause dehydration (24), while obesity can disrupt fluid distribution, resulting in elevated serum sodium levels (25). Therefore, participants with blood glucose levels > 140 mg/dL or a BMI > 35 kg/m^2^ were excluded. Since systolic blood pressure and cholesterol were used to calculate biological age, participants taking antihypertensive or lipid-lowering medications were excluded to avoid potential confounding. After applying these exclusion criteria, the final study cohort comprised 18,301 eligible participants (Figure 1).

### 2.2 Serum sodium measurement

Serum sodium levels were extracted from the NHANES 1999–2018 standard biochemistry files. Sodium concentration in biological fluids was measured using the Beckman LX and DxC systems with the indirect (or diluted) I.S.E. method. Serum sodium concentrations are quantified in units of millimoles per liter (mmol/L). Participants were categorized into four quartiles based on serum sodium levels: 135–138 mmol/L, 138–139 mmol/L, 139–141 mmol/L, and 141–145 mmol/L.

### 2.3 Calculation of biological age and ∆age

Biological age was computed via the Klemera and Doubal method (Equations 1–4) (26), a widely used approach for estimating biological age that leverages multiple biomarkers to provide a comprehensive assessment of aging across different organ systems. Eight biomarkers, representing diverse organ systems and physiological processes, are integrated into this method: cardiovascular (systolic blood pressure), renal (blood urea nitrogen, creatinine), metabolic (total cholesterol, glycated hemoglobin, alkaline phosphatase), and immune/inflammatory (CRP, albumin).

(1) $$BA_{E}=\frac{\sum_{j=1}^{m}\left(x_{j}-q_{j}\right)\left(\frac{k_{j}}{s_{j}^{2}}\right)}{\sum_{j=1}^{m}{\left(\frac{k_{j}}{s_{j}}\right)}^{2}}$$

(2) $$r_{\mathrm{char}}=\frac{\sum_{j=1}^{m}\frac{r_{j}^{2}}{\sqrt{1-r_{j}^{2}}}}{\sum_{j=1}^{m}\frac{r_{j}}{\sqrt{1-r_{j}^{2}}}}$$

(3) $$\begin{array}{l} s_{\mathrm{BA}}^{2}=\frac{\sum_{j=1}^{n}{\left(\left(BA_{Ei}-CA_{i}\right)-\frac{\sum_{i=1}^{n}\left(BA_{Ei}-CA_{i}\right)}{n}\right)}^{2}}{n}-\\ \left(\frac{1-r_{\mathrm{char}}^{2}}{r_{\mathrm{char}}^{2}}\right)\times \left(\frac{{\left(CA_{\max-}CA_{\text{min}}\right)}^{2}}{12m}\right) \end{array}$$

(4) $$\mathrm{Biological}\;\mathrm{age}=\frac{\sum_{j=1}^{m}\left(x_{j}-q_{j}\right)\left(\frac{k_{j}}{s_{j}^{2}}\right)+\frac{CA}{s_{\mathrm{BA}}^{2}}}{\sum_{j=1}^{m}{\left(\frac{k_{j}}{s_{j}^{2}}\right)}^{2}+\frac{1}{s_{\mathrm{BA}}^{2}}}$$

In this notation, *j* and *i* indicate the total number of biomarkers and samples, respectively. For each biomarker *j*, the regression coefficients *q* (intercept), *k* (slope), and *s* (root mean square error, RMSE) were calculated by modeling its relationship with chronological age. *CA* denotes chronological age (true age), while $r_{j}^{2}$ represents the variance explained by regressing chronological age on each biomarker.

We also calculated ∆age (biological age—chronological age), where a positive value indicates that an individual is physiologically older than their chronological age, while a negative value suggests a younger physiological age. This metric provides a quantitative measure of the discrepancy between biological and chronological aging.

### 2.4 Covariates

To control potential confounding variables associated with serum sodium levels (as a surrogate of hydration status) and biological aging, we incorporated a comprehensive set of covariates into the analysis. Covariates included demographic factors (age, gender, race/ethnicity, education level, marital status, income-to-poverty ratio), lifestyle factors (tobacco use, alcohol consumption), health conditions (diabetes, kidney conditions, cancers), and clinical measures (triglycerides, body mass index [BMI]). These covariates were incorporated into the regression models as potential confounders.

Race/ethnicity was categorized into non-Hispanic White, non-Hispanic Black, Mexican American, and other/multiracial groups. Education level was classified into three tiers: less than high school, high school or equivalent, and post-secondary education. Marital status was grouped as married/cohabiting, widowed/divorced/separated, and never married. Tobacco and alcohol use were treated as binary variables (yes/no). Diabetes, kidney conditions, and cancers were ascertained based on clinical diagnoses by physicians or healthcare professionals.

### 2.5 Statistical analysis

Statistical analyses were carried out with R software (version 4.2) and EmpowerStats (version 4.1). Continuous variables were described using means and standard deviations, whereas categorical variables were summarized as frequencies with percentages. To compare demographic characteristics across the four serum sodium quartiles, χ2 tests were applied to categorical variables, and one-way ANOVA was used for continuous variables.

Weighted multivariate linear regression models were used to assess the linear associations between serum sodium levels and biological age/∆age, accounting for the complex sampling design of NHANES. The crude model was unadjusted; the second model accounted for age, sex, and race; and the third model extended adjustments to include education, marital status, PIR, tobacco use, alcohol consumption, diabetes, weak/failing kidneys, cancer, triglyceride and BMI. For ∆age models, chronological age was not included as a covariate since ∆age is derived from it (biological age – chronological age). Generalized additive models with smoothed curve fitting were applied to assess nonlinear associations between serum sodium and biological age/∆age. Furthermore, threshold effect analysis was used to examine the dose–response relationship and identify potential turning points. Subgroup and interaction analyses assessed serum sodium and biological age/∆age relationships across populations. Significance was set at *p* < 0.05 (two-tailed).

## 3 Results

### 3.1 Participant characteristics

This study included 18,301 participants, with an average age of 43.11 ± 16.73 years and 52.23% (9,558) being male. The mean serum sodium level was 139.36 ± 1.98 mmol/L, and the average biological age was 42.55 ± 16.17 years. Stratified by serum sodium quartiles, 18.14% of participants were in the lowest quartile (135–138 mmol/L), while 27.12% were in the highest quartile (141–145 mmol/L). Table 1 presents the baseline characteristics of participants stratified by serum sodium quartiles (Q1: <138, Q2: 138–139, Q3: 139–141, Q4: ≥141 mmol/L). Statistically significant differences were observed across quartiles for age, sex, race/ethnicity, and prevalence of chronic conditions (*p* < 0.001). Notably, participants in the highest quartile (Q4) were older, more likely to be male, and had higher rates of diabetes and cancer compared to those in the lowest quartile (Q1). These variations underscore the importance of adjusting for these covariates in subsequent analyses.

**Table 1:** Basic characteristics of participants by serum sodium range (mmol/L).

Characteristics	Serum sodium quartile (mmol/L)				*p* value
	Q1 (<138) *N* = 3,320	Q2 (138–139) *N* = 2,978	Q3 (139–141) *N* = 7,040	Q4 (≥141) *N* = 4,963	
Age (years)	41.75 ± 15.84	41.49 ± 15.69	42.37 ± 16.45	46.04 ± 17.92	<0.001
Gender, (%)					<0.001
Male	1,475 (44.43%)	1,448 (48.62%)	3,751 (53.28%)	2,884 (58.11%)	
Female	1845 (55.57%)	1,530 (51.38%)	3,289 (46.72%)	2079 (41.89%)	
Race/ethnicity, (%)					<0.001
Non-Hispanic White	1,501 (45.21%)	1,282 (43.05%)	3,252 (46.19%)	2,343 (47.21%)	
Non-Hispanic Black	527 (15.87%)	487 (16.35%)	1,167 (16.58%)	880 (17.73%)	
Mexican American	753 (22.68%)	663 (22.26%)	1,477 (20.98%)	964 (19.42%)	
Other race/multiracial	539 (16.23%)	546 (18.33%)	1,144 (16.25%)	776 (15.64%)	
Education, (%)					0.814
Under high school	875 (26.36%)	787 (26.43%)	1806 (25.65%)	1,295 (26.09%)	
High school or equivalent	765 (23.04%)	654 (21.96%)	1,620 (23.01%)	1,158 (23.33%)	
Above high school	1,680 (50.60%)	1,537 (51.61%)	3,614 (51.34%)	2,510 (50.57%)	
Marital status, (%)					0.003
Married/cohabiting	2013 (60.63%)	1793 (60.21%)	4,377 (62.17%)	2,978 (60.00%)	
Widowed/divorced/separated	571 (17.20%)	481 (16.15%)	1,101 (15.64%)	910 (18.34%)	
Never married	736 (22.17%)	704 (23.64%)	1,562 (22.19%)	1,075 (21.66%)	
PIR	2.55 ± 1.56	2.54 ± 1.56	2.60 ± 1.58	2.58 ± 1.56	0.148
Tobacco use, (%)					0.001
Current users	1817 (54.73%)	1741 (58.46%)	3,894 (55.31%)	2,678 (53.96%)	
No current users	1,503 (45.27%)	1,237 (41.54%)	3,146 (44.69%)	2,285 (46.04%)	
Alcohol consumption, (%)					<0.001
Yes	2,792 (84.10%)	2,516 (84.49%)	5,912 (83.98%)	4,037 (81.34%)	
No	528 (15.90%)	462 (15.51%)	1,128 (16.02%)	926 (18.66%)	
Diabetes, (%)					0.002
Yes	57 (1.72%)	57 (1.91%)	145 (2.06%)	140 (2.82%)	
No	3,236 (97.47%)	2,897 (97.28%)	6,846 (97.24%)	4,767 (96.05%)	
Borderline	27 (0.81%)	24 (0.81%)	49 (0.70%)	56 (1.13%)	
Weak/failing kidneys, (%)					0.562
Yes	32 (0.96%)	32 (1.07%)	74 (1.05%)	63 (1.27%)	
No	3,288 (99.04%)	2,946 (98.93%)	6,966 (98.95%)	4,900 (98.73%)	
Cancer, (%)					<0.001
Yes	186 (5.60%)	140 (4.70%)	371 (5.27%)	349 (7.03%)	
No	3,134 (94.40%)	2,838 (95.30%)	6,669 (94.73%)	4,614 (92.97%)	
Triglyceride (mg/dL)	133.77 ± 115.55	138.01 ± 118.94	131.84 ± 104.75	133.23 ± 97.85	0.073
BMI (kg/m^2^)	26.22 ± 4.17	26.26 ± 4.18	26.22 ± 4.16	26.19 ± 4.15	0.912
Biological age (years)	41.28 ± 15.25	40.92 ± 15.20	41.79 ± 15.89	45.44 ± 17.31	<0.001
∆age (years)	−0.47 ± 3.57	−0.57 ± 3.51	−0.58 ± 3.56	−0.59 ± 3.87	0.459

Mean ± SD or Median (IQR) for continuous variables: the *P* value was calculated by one-way ANOVA. *N* (%) for categorical variables: the *P* value was calculated by chi-square test. Q Quartile, PIR the ratio of income to poverty, BMI Body mass index, ∆age = biological age—chronological age.

### 3.2 Association between serum sodium and biological age and ∆age

Table 2 summarizes the results of multivariable regression analyses assessing the linear association between serum sodium and biological aging. In the unadjusted model (Model 1), each 1 mmol/L increase in serum sodium was associated with a significant 0.83-year increase in biological age (95% CI: 0.71–0.95). However, after adjusting for demographic and lifestyle covariates (Models 2 and 3), this association attenuated and became non-significant. For ∆age, a consistent negative association was observed in both adjusted models, with each 1 mmol/L increase in serum sodium associated with a 0.04–0.06 year decrease in ∆age, suggesting that higher serum sodium may be linked to slower biological aging relative to chronological age.

**Table 2:** Logistic regression analysis for the relationship between serum sodium (mmol/L) and biological aging.

Serum sodium quartiles	Crude model *β* (95% CI)	Model 2 *β* (95% CI)	Model 3 *β* (95% CI)
Biological age (continuous)	0.83 (0.71, 0.95)	−0.01 (−0.04, 0.01)	−0.00 (−0.03, 0.02)
Biological age (quartile)			
Quartile 1	Reference	Reference	Reference
Quartile 2	−0.36 (−1.15, 0.44)	−0.19 (−0.36, −0.02)	−0.21 (−0.37, −0.05)
Quartile 3	0.51 (−0.15, 1.17)	−0.22 (−0.36, −0.08)	−0.19 (−0.33, −0.06)
Quartile 4	4.17 (3.46, 4.87)	−0.11 (−0.26, 0.04)	−0.08 (−0.22, 0.07)
*P* for trend	<0.001	0.163	0.415
∆age (continuous)	−0.02 (−0.05, 0.01)	−0.06 (−0.08, −0.03)	−0.04 (−0.07, −0.02)
∆age (quartile)			
Quartile 1	Reference	Reference	Reference
Quartile 2	−0.10 (−0.28, 0.08)	−0.18 (−0.35, −0.00)	−0.23 (−0.39, −0.06)
Quartile 3	−0.11 (−0.26, 0.04)	−0.25 (−0.40, −0.10)	−0.23 (−0.37, −0.09)
Quartile 4	−0.12 (−0.28, 0.04)	−0.35 (−0.50, −0.19)	−0.28 (−0.42, −0.13)
*P* for trend	0.161	<0.001	<0.001

Data are presented as β, 95% confidence intervals. Crude model: no covariates were adjusted. Model 2: age, gender and race were adjusted. Model 3: age, gender, race, education, marital status, PIR, tobacco use, alcohol consumption, diabetes, weak/failing kidneys, cancer, triglyceride and BMI were adjusted. ∆age adjusted for all covariates except for age. ∆age = biological age—chronological age.

**Figure 2:**
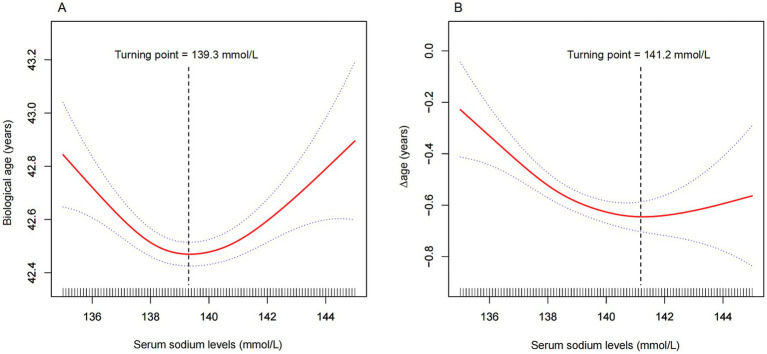
Nonlinear association between serum sodium and **(A)** biological age, and **(B)** ∆age. The solid red line represents the smooth curve fit between variables, and the blue band indicates the 95% confidence interval. Turning points were identified at 139.3 mmol/L for biological age **(A)** and 141.2 mmol/L for ∆age **(B)**. Models were adjusted for age, sex, race, education, marital status, income-to-poverty ratio, tobacco use, alcohol consumption, diabetes, kidney disease, cancer, triglycerides, and BMI. For ∆age, chronological age was excluded from adjustment.

As shown in Figure 2A and Table 3, a significant U-shaped nonlinear association was identified between serum sodium and biological age. Biological age declined with increasing sodium levels up to the inflection point (139.3 mmol/L), beyond which it began to rise again. Below this threshold, each 1 mmol/L increase in serum sodium was associated with a reduction of 0.10 years in biological age (95% CI: −0.15, −0.05), while above the threshold, the increase was 0.08 years (95% CI: 0.04, 0.13).

**Table 3:** Threshold effect analysis of serum sodium (mmol/L) on biological age (years)/∆age (years) using piece-wise linear regression.

Serum sodium threshold (mmol/L)	Crude *β* (95% CI) *P* value	Adjusted *β* (95% CI) *P* value
Biological age		
Serum sodium < 139.3 mmol/L	−0.04 (−0.28, 0.21) 0.770	−0.10 (−0.15, −0.05) < 0.001
Serum sodium ≥ 139.3 mmol/L	1.60 (1.38, 1.82) < 0.001	0.08 (0.04, 0.13) < 0.001
∆age		
Serum sodium < 141.2 mmol/L	−0.04 (−0.07, −0.00) 0.034	−0.07 (−0.10, −0.04) < 0.001
Serum sodium ≥ 141.2 mmol/L	0.06 (−0.04, 0.16) 0.217	0.08 (−0.01, 0.17) 0.081

Crude: no adjustment. Adjusted: biological age adjusted for age, gender, race, education, marital status, PIR, tobacco use, alcohol consumption, diabetes, weak/failing kidneys, cancer, triglyceride and BMI. ∆age adjusted for all covariates except for age. ∆age = biological age—chronological age.

Similarly, for ∆age, Figure 2B and Table 3 reveal that sodium levels below 141.2 mmol/L were significantly associated with a lower ∆age, indicating a younger biological profile relative to chronological age. Beyond 141.2 mmol/L, this association was no longer statistically significant.

**Figure 3:**
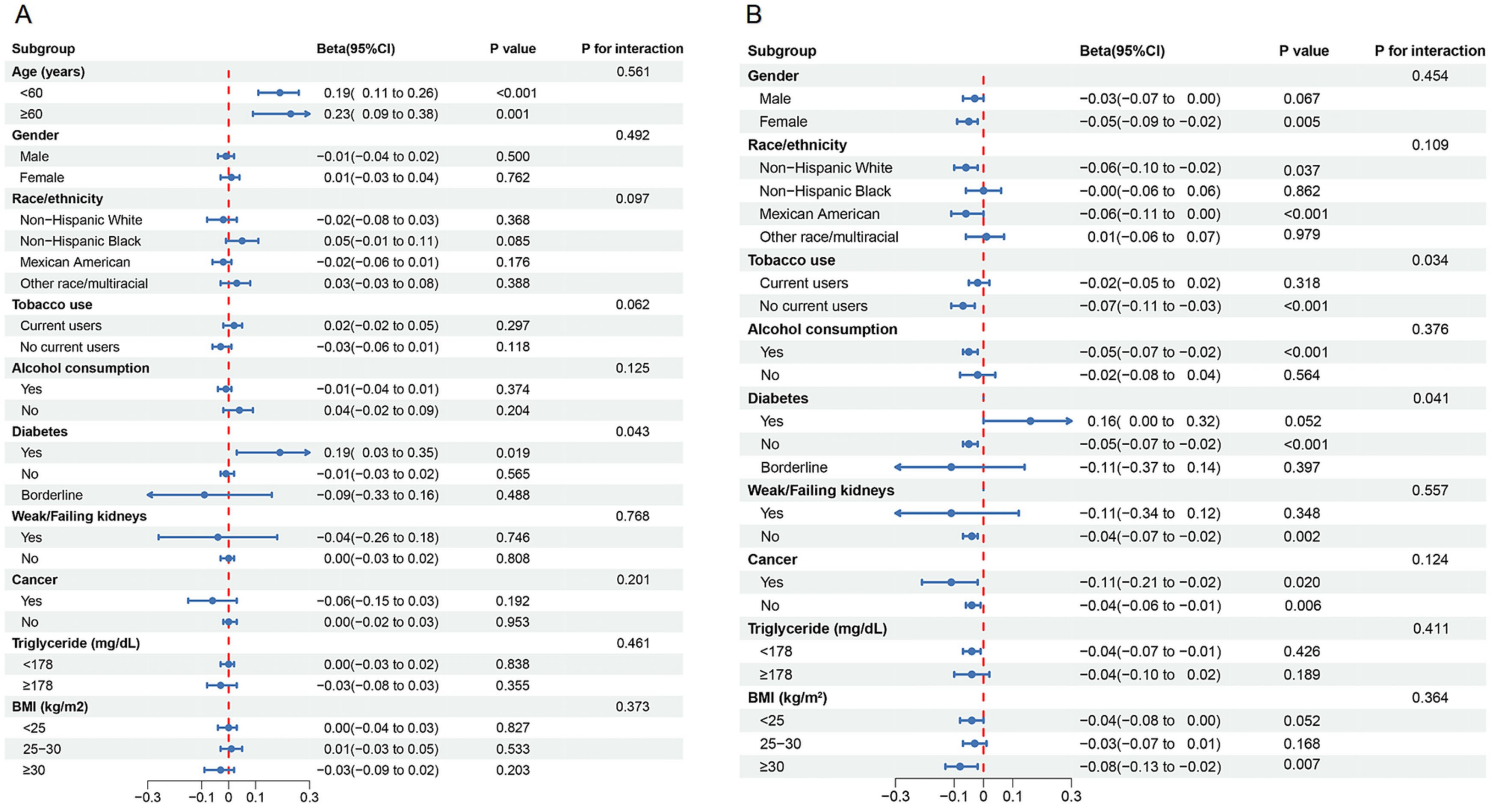
Subgroup analysis for the association between serum sodium and **(A)** biological age, and **(B)** Δage.

Subgroup analyses illustrated in Figure 3 further support the robustness of the findings. Figure 3A indicates that the U-shaped association between serum sodium and biological age varied across subgroups, with significant modification observed in individuals with diabetes (*P* for interaction = 0.043). In Figure 3B, the inverse association between serum sodium and ∆age was consistent across most subgroups. Interestingly, in diabetic individuals, the association reversed (*β* = 0.16), suggesting greater biological aging with increasing sodium levels. Tobacco use also significantly modified the association with ∆age (*P* for interaction = 0.034), supporting the hypothesis that specific high-risk populations may be more sensitive to serum sodium variations.

## 4 Discussion

A significant dose–response relationship between serum sodium and biological age was observed in this cross-sectional study of 18,301 participants. Notably, this study is the first to report a U-shaped relationship between serum sodium and biological age after controlling for confounding factors. Biological age reached its lowest point when serum sodium levels were around 138–142 mmol/L (Figure 2A). Meanwhile, our findings demonstrated a negative correlation between serum sodium levels and ∆age, which remained consistent across almost all subgroups. This suggests that elevated serum sodium levels are associated with a younger biological age. However, this negative correlation reached a threshold when serum sodium levels exceeded 141.2 mmol/L (Figure 2B). These results indicate that maintaining optimal serum sodium levels may help delay the biological aging process.

Age-related degenerative changes are key contributing factors to the development of most chronic diseases. Previous studies have shown that elevated serum sodium concentrations (> 144 mmol/L) are associated with increased incidence and mortality risk among women aged over 50, likely due to dehydration-induced hypernatremia (27). Conversely, community-based subjects with serum sodium levels at the lower end of the normal range (135–137 mmol/L) exhibit higher mortality and cardiovascular disease incidence, which may be attributed to conditions causing electrolyte imbalances (28). These findings are consistent with our study’s conclusions, indicating that both elevated (> 142 mmol/L) and reduced (< 138 mmol/L) serum sodium levels may contribute to accelerated aging. Maintaining an optimal hydration homeostasis over time could potentially slow down the aging process, as well as the chronic diseases and mortality associated with aging.

The cohort study by Natalia et al. (20) demonstrated that the risk of chronic diseases and mortality increases regardless of whether serum sodium levels fall within the lower range (< 137 mmol/L) or the higher range (> 142 mmol/L). When investigating the relationship between serum sodium and biological age, their study indicates that even mild hypernatremia (> 142 mmol/L) may accelerate vascular endothelial dysfunction, thereby promoting biological aging. However, participants with serum sodium levels below 137 mmol/L did not exhibit a similar association. In the study cohort of 11,255 individuals, only 122 participants had serum sodium levels below 137 mmol/L. This subgroup was notably smaller compared to other serum sodium groups, resulting in wide confidence intervals. This may explain the inconsistency between the association of lower-range serum sodium levels with biological age and the outcomes related to chronic disease and mortality risk. In our study, which included 18,301 participants, 1,398 individuals had serum sodium levels below 137 mmol/L. This larger sample size provides a more robust representation of the aging status among participants in the lower serum sodium range.

To further explore the robustness of the U-shaped relationship, we conducted stratified analyses based on key demographic and health characteristics. In the stratified analysis of biological age, this association exhibit differences among diabetic population. This phenomenon may be attributed to the increased susceptibility of diabetic patients to dehydration or electrolyte imbalances, resulting from osmotic diuresis, undiagnosed or inadequately managed conditions, contributing factors, or the use of certain antidiabetic medications (29). Therefore, adequate intake of water and fluids with appropriate electrolyte composition is crucial for preventing dehydration in this population. In a one-week human water intervention trial involving an additional daily water intake of 3 liters, approximately one-third of participants exhibited a reduction in copeptin levels (a marker of vasopressin) after increased water intake (30). High copeptin levels will increase cardiovascular disease and premature mortality risk in diabetic patients, suggesting that optimal hydration status may improve adverse outcomes by enhancing glucose metabolism (31). This may also explain why the negative correlation between serum sodium and ∆age shows significant variations between diabetic and non-diabetic patients. Smoking is known to significantly accelerate the aging process (32). Our findings indicate that the negative correlation between serum sodium and ∆age was not pronounced in smoking participants. Therefore, we recommend that individuals who smoke, have diabetes, or exhibit a biological age older than their chronological age, along with suboptimal serum sodium levels, should pay greater attention to hydration management.

The U-shaped relationship observed in this study may reflect the complex physiological responses elicited by deviations from optimal serum sodium levels. From a mechanistic perspective, both hypo- and hypernatremia, even within the clinically normal range, can trigger osmoregulatory stress at the cellular level. Hypohydration-induced hypertonicity elevates intracellular sodium concentrations, leading to increased vasopressin release and cellular shrinkage, which can stimulate pro-aging pathways such as oxidative stress and mitochondrial dysfunction (33, 34). Conversely, low-normal sodium levels may reflect underlying conditions like chronic inflammation, subclinical illness, or dilutional hyponatremia, each of which have been independently associated with frailty and biological aging (35, 36). Sodium imbalance also affects protein folding and degradation pathways (proteostasis), disrupts autophagy, and promotes inflammasome activation, contributing to cellular senescence (34, 37). These physiological alterations provide a plausible biological basis for our findings and support the notion that maintaining optimal hydration and sodium homeostasis may play a role in modulating the pace of biological aging.

Several limitations should be acknowledged. First, the cross-sectional design precludes causal inference between serum sodium levels and biological aging. Serum sodium was measured at a single time point, which may not reflect long-term hydration status. Although serum sodium is a practical hydration marker in large-scale studies, more direct measures—such as plasma osmolality and urine specific gravity—were not consistently available across the full NHANES cycles analyzed, and thus could not be included. Additionally, important confounders such as dietary sodium intake and total water consumption were excluded due to inconsistent availability across survey years and the limitations of 24-h recall methods, which are subject to recall bias and may not accurately reflect absorption or physiological balance. These variables should be considered in future studies employing more comprehensive dietary and hydration assessments. Despite these limitations, our study has notable strengths. It is the first to examine the association between serum sodium levels and biological aging in a nationally representative U.S. adult cohort. The large and diverse sample enabled robust subgroup analyses, enhancing the generalizability of our findings across different populations.

From a clinical and public health perspective, these findings underscore the potential utility of monitoring serum sodium levels—not only to assess hydration status, but also as an indirect indicator of biological aging. Since serum sodium is a routinely measured biomarker in clinical practice, it could be integrated into screening programs to identify individuals at risk of accelerated aging, especially among vulnerable populations such as smokers and patients with diabetes. Maintaining serum sodium within an optimal range (138–142 mmol/L) may serve as a simple, cost-effective target for hydration management aimed at promoting healthy aging. These insights may inform future updates to hydration guidelines and preventive care strategies, particularly as the global population continues to age.

## 5 Conclusion

The study demonstrated that both lower (< 137 mmol/L) and higher (> 142 mmol/L) serum sodium levels, despite falling within the normal range, are associated with faster biological aging. Our findings indicate that maintaining optimal serum sodium levels could contribute to delaying biological aging. Future intervention-based randomized controlled trials should aim to establish causality and explore the mechanisms through which optimal serum sodium levels modulate biological aging.

## Data Availability

The original contributions presented in the study are included in the article/supplementary material, further inquiries can be directed to the corresponding author.
